# Synthesis and spectroscopic and structural characterization of spiro­[indoline-3,3′-indolizine]s formed by 1,3-dipolar cyclo­additions between isatins, pipecolic acid and an electron-deficient alkene

**DOI:** 10.1107/S2053229621007142

**Published:** 2021-08-06

**Authors:** Pablo E. Romo, Jairo Quiroga, Justo Cobo, Christopher Glidewell

**Affiliations:** a Grupo de Investigación de Compuestos Heterocíclicos, Departamento de Química, Universidad del Valle, A.A. 25360 Cali, Colombia; b Departamento de Química Inorgánica y Orgánica, Universidad de Jaén, 23071 Jaén, Spain; c School of Chemistry, University of St Andrews, Fife, KY16 9ST, United Kingdom

**Keywords:** synthesis, heterocycle, spiro­[indoline-3,3′-indolizine], NMR spectroscopy, reaction mechanism, crystal structure, stereochemistry, mol­ecular conformation, supra­molecular assembly

## Abstract

Five new spiro­[indoline-3,3′-indolizine]s have been synthesized with high regio- and stereo­specificity in one-pot three-com­ponent reactions between a sub­sti­tuted indole-2,3-dione, (*S*)-pipecolic acid and *trans*-3-benzoyl­acrylic acid, and subsequently characterized using a combination of elemental analysis, IR and ^1^H and ^13^C NMR spectroscopy, mass spectrometry and crystal structure analysis. (1′*SR*,2′*SR*,3*RS*,8a′*RS*)-2′-Benzoyl-5-fluoro-2-oxo-1′,5′,6′,7′,8′,8a′-hexa­hydro-2′*H*-spiro­[indoline-3,3′-indolizine]-1′-carb­oxy­lic acid, C_23_H_21_FN_2_O_4_, (I), and (1′*SR*,2′*SR*,3*RS*,8a′*RS*)-2′-benzoyl-5-methyl-2-oxo-1′,5′,6′,7′,8′,8a′-hexa­hy­dro-2′*H*-spiro[indoline-3,3′-indolizine]-1′-carb­oxy­lic acid, C_24_H_24_N_2_O_4_, (II), are isomorphous, as are (1′*SR*,2′*SR*,3*RS*,8a′*RS*)-2′-benzoyl-1-methyl-2-oxo-1′,5′,6′,7′,8′,8a′-hexa­hydro-2′*H*-spiro­[indoline-3,3′-indolizine]-1′-carb­oxy­lic acid, C_24_H_24_N_2_O_4_, (III), and (1′*SR*,2′*SR*,3*RS*,8a′*RS*)-2′-benzoyl-5-chloro-1-methyl-2-oxo-1′,5′,6′,7′,8′,8a′-hexa­hydro-2′*H*-spiro­[indoline-3,3′-indolizine]-1′-carb­oxy­lic acid, C_24_H_23_ClN_2_O_4_, (IV). Within each isomorphous pair, the spiro ring systems show some conformational differences. In each of (I) and (II), the mol­ecules are linked into com­plex sheets by a combination of four types of hydrogen bond, and in each of (III) and (IV), a combination of O—H⋯O and C—H⋯π(arene) hydrogen bonds links the mol­ecules to form a chain of centrosymmetric rings. In (1′*SR*,2′*SR*,3*RS*,8a′*RS*)-2′-benzoyl-1-hexyl-2-oxo-1′,5′,6′,7′,8′,8a′-hexa­hydro-2′*H*-spiro­[indoline-3,3′-indolizine]-1′-carb­oxy­lic acid, C_29_H_34_N_2_O_4_, (V), a combination of five hydrogen bonds links the mol­ecules into sheets of alternating *R*_2_^2^(16) and *R*_6_^6^(46) rings. A mechanism is proposed for the formation of com­pounds (I)–(V) and some com­parisons with related structures are made.

## Abstract 2

New spiro­[indoline-3,3′-indolizine]s have been synthesized with high regio- and stereo­specificity from simple starting materials in a one-step process. Different combinations of hydrogen bonds link the mol­ecules to form either sheets or chains of rings.

## Introduction   

Spirooxindoles are a privileged category of heterocycles con­taining a unique and versatile scaffold for novel drug dis­covery in fields as diverse as analgesics, anti­cancer, anti-inflammatory and anti­microbial agents, and anti­oxidants, whose structure–activity relationships and mol­ecular mechanisms of action have recently been reviewed (Zhou *et al.*, 2020 ▸).

Multicom­ponent reactions can provide versatile and efficient routes to new heterocyclic systems, permitting the incor­poration of a wide variety of functionalities by the com­bination of three or more simple building blocks (Dömling, 2002 ▸; Hulme & Gore, 2003 ▸; Orru & de Greef, 2003 ▸; Quiroga *et al.*, 2007 ▸, 2014 ▸). The spirooxindole core is readily obtained using 1,3-dipolar cycloadditions between electron-deficient alkenes and an azomethine ylide, generated *in situ* from an isatin (indole-2,3-dione) and an amino acid (Grigg *et al.*, 1984 ▸; Al-Majid *et al.*, 2020 ▸; Ghosh *et al.*, 2020 ▸). We have recently reported the regio- and stereo­specific synthesis, spec­troscopic characterization and crystal structures of some spiro­[indoline-3,3′-pyrrolizine]s (Quiroga *et al.*, 2017 ▸) and di­spiro­[indoline-3,3′-pyrrolizine-1′,5′-thia­zolidine]s (Romo *et al.*, 2020 ▸), formed in a single step from mixtures of a substituted isatin, a cyclic amino com­pound and an electron-deficient alkene. As a development of these previous studies, we have now investigated the reactions between isatins, pipecolic acid [(*RS*)-piperidine-2-carboxylic acid] and *trans*-3-benzoyl­acrylic acid [(*E*)-4-oxo-4-phenyl­but-2-enoic acid] to form spiro­[indoline-3,3′-indolizine]s. Here we report the synthesis and spectroscopic charaterization, and the mol­ecular and supra­molecular structures of five representative examples, namely, (1′*SR*,2′*SR*,3*RS*,8a′*RS*)-2′-benzoyl-5-fluoro-2-oxo-1′,5′,6′,7′,8′,8a′-hexa­hydro-2′*H*-spiro­[indoline-3,3′-indolizine]-1′-carb­oxy­lic acid, (I), (1′*SR*,2′*SR*,3*RS*,8a′*RS*)-2′-benzoyl-5-methyl-2-oxo-1′,5′,6′,7′,8′,8a′-hexa­hydro-2′*H*-spiro­[indoline-3,3′-indolizine]-1′-carb­ox­y­lic acid, (II), (1′*SR*,2′*SR*,3*RS*,8a′*RS*)-2′-benzoyl-1-methyl-2-oxo-1′,5′,6′,7′,8′,8a′-hexa­hydro-2′*H*-spiro­[indoline-3,3′-indolizine]-1′-carb­oxy­lic acid, (III), (1′*SR*,2′*SR*,3*RS*,8a′*RS*)-2′-ben­zoyl-5-chloro-1-methyl-2-oxo-1′,5′,6′,7′,8′,8a′-hexa­hydro-2′*H*-spiro­[indoline-3,3′-indolizine]-1′-carb­oxy­lic acid, (IV), and (1′*SR*,2′*SR*,3*RS*,8a′*RS*)-2′-benzoyl-1-hexyl-2-oxo-1′,5′,6′,7′,8′,8a′-hexa­hydro-2′*H*-spiro­[indoline-3,3′-indolizine]-1′-carb­oxy­lic acid, (V) (Scheme 1).
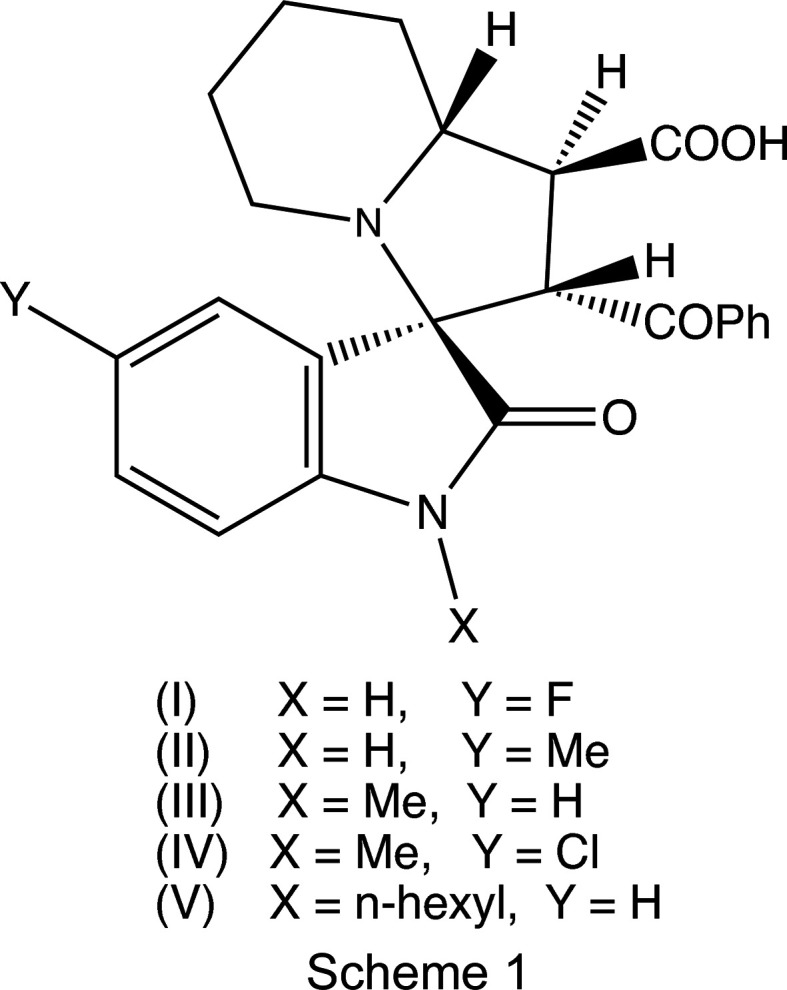


**Figure 1:**
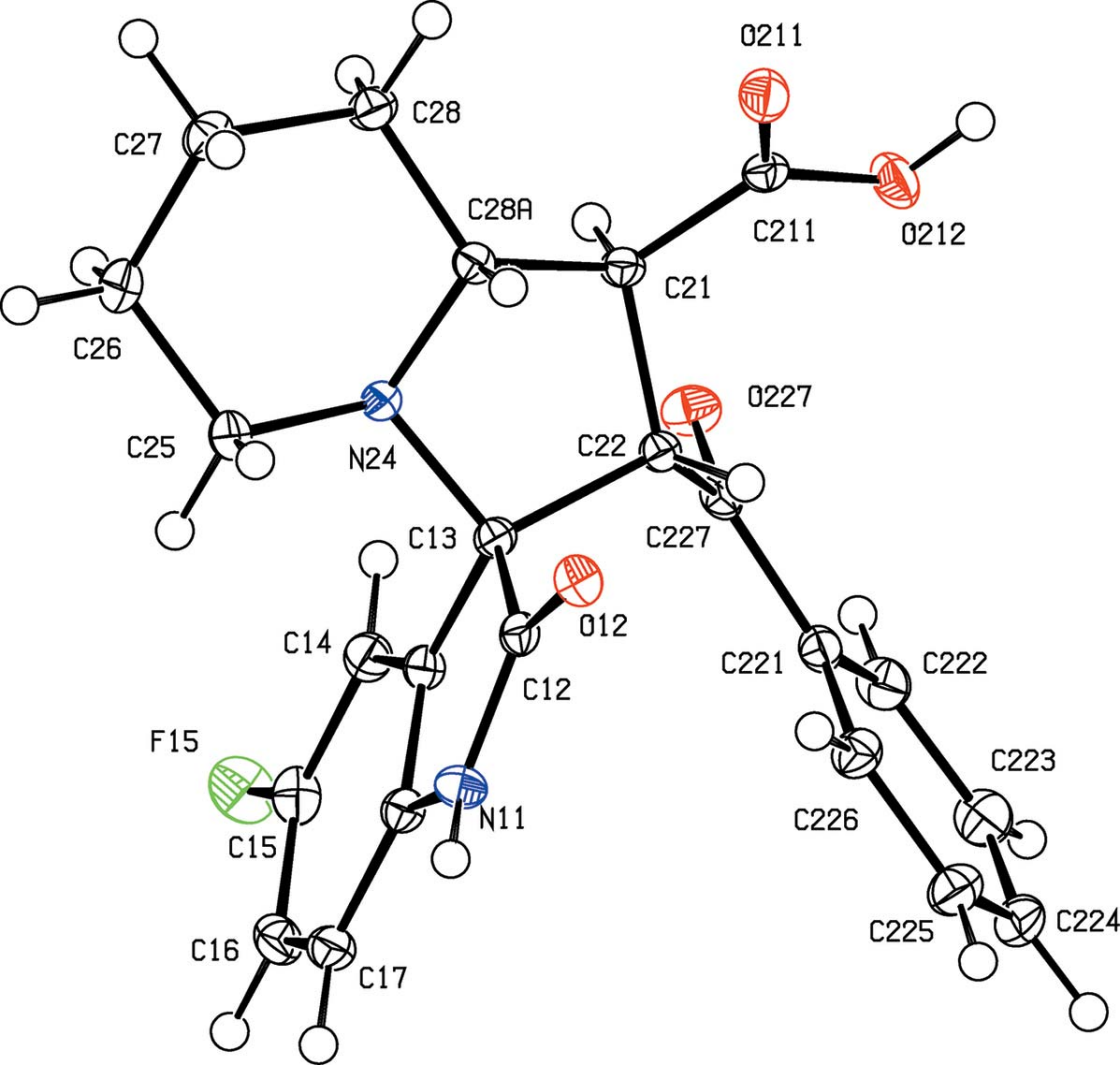
The mol­ecular structure of the (1′*S*,2′*S*,3*RS*,8a′*R*) enanti­omer of compound (I), showing the atom-labelling scheme. Displacement ellipsoids are drawn at the 50% probability level.

Compounds (I)–(V) were formed in yields between 48 and 69% in one-pot reactions between an appropriately substituted isatin (see Scheme 2), pipecolic acid acting as the cyclic amine com­ponent and *trans*-3-benzoyl­acrylic acid acting as the electron-deficient alkene to give the products defined in Scheme 1 and Figs. 1 ▸–5 ▸ ▸ ▸ ▸. Products (I)–(V) were all isolated as single racemic stereoisomers and all have been characterized by a combination of elemental analysis, IR and ^1^H and ^13^C NMR spectroscopy, mass spectrometry and X-ray crystal structure analysis, which enables a com­plete definition of the stereochemistry.

## Experimental   

### Synthesis and crystallization   

All reagents and solvents were obtained commercially and all were used as recieved. For the synthesis of com­pounds (I)–(V), mixtures of pipecolic acid (64.6 mg, 0.5 mmol), the appropriately substituted isatin (0.5 mmol) [5-fluoro­isatin (83.5 mg) for (I), 5-methyl­isatin (80.6 mg) for (II), 1-methyl­isatin (80.6 mg) for (III), 5-chloro-1-methyl­isatin (97.8 mg) for (IV) and 1-hexyl­isatin (115.6 mg) for (V)] and *trans*-3-ben­zoyl­acrylic acid [(*E*)-4-oxo-4-phenyl­but-2-enoic acid] (88.1 mg, 0.5 mmol) in aceto­nitrile (10 ml) were heated under reflux until the reactions were com­plete, as judged by thin-layer chromatography (TLC) monitoring (reactions times were all in the range 8–12 h). The reaction mixtures were allowed to cool to ambient temperature, giving the crystalline products (I)–(V), which were collected by filtration and then dried in air. No further purification was required, as judged by TLC and spectroscopic examination, and crystals suitable for single-crystal X-ray diffraction were, in each case, selected directly from the synthesized samples.
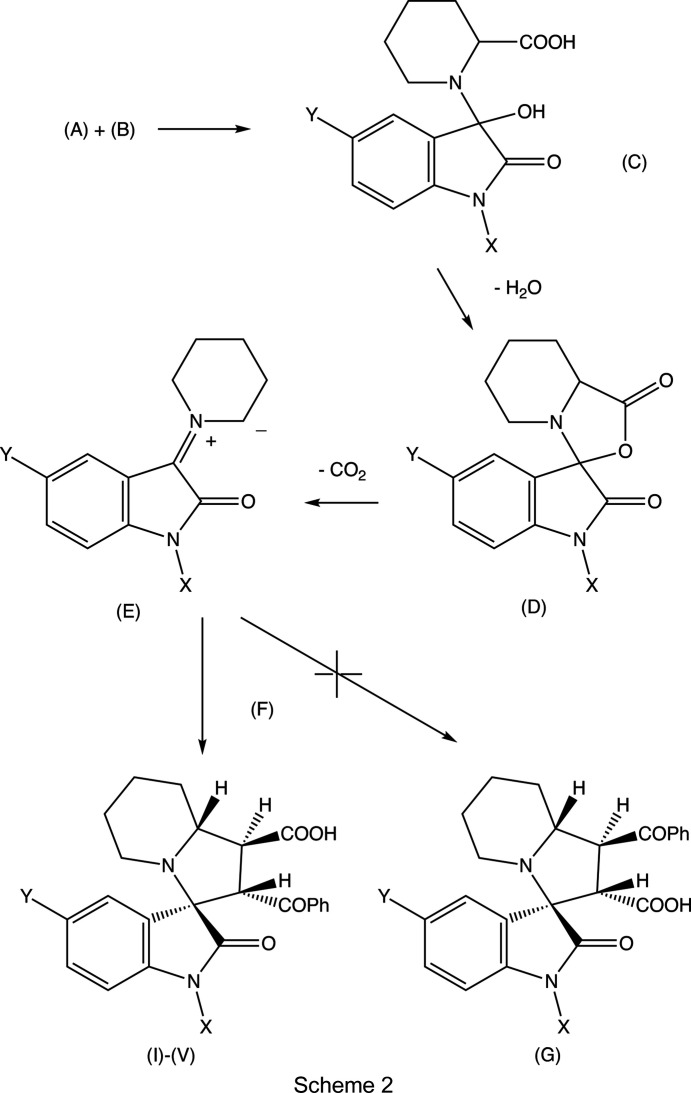


Compound (I): yield 68%; m.p. 508–509 K. Analysis found (%): C 67.6, H 5.2, N 6.8; calculated for C_23_H_21_FN_2_O_4_ (%): C 67.6, H 5.2, N 6.9. FT–IR (ATR, cm^−1^): 3478, 3096, 2937, 1715, 1676. NMR (DMSO-*d*_6_): δ(^1^H) 1.11–1.23 (*m*, 2H, H8′, H7′), 1.23–1.33 (*m*, 1H, H6′), 1.42–1.54 (*m*, 1H, H7′), 1.66–1.79 (*m*, 1H, H8′), 2.05–2.19 (*m*, 2H, H5′, H6′), 2.20–2.32 (*m*, 1H, H5′), 3.25 (*t*, *J* = 10.0 Hz, 1H, H8a′), 3.45 (*t*, *J* = 9.7 Hz, 1H, H1′), 4.50 (*d*, *J* = 9.7 Hz, 1H, H2′), 6.41 (*dd*, *J* = 8.6, 4.3 Hz, 1H), 6.54 (*dd*, *J* = 8.2, 2.7 Hz, 1H), 6.78 (*td*, *J* = 9.0, 2.7 Hz, 1H), 7.32 (*t*, *J* = 7.6 Hz, 2H), 7.40–7.51 (*m*, 3H), 10.52 (*s*, 1H, NH), 12.69 (*s*, 1H, COOH); δ(^13^C) 23.7 (C8′), 25.5 (C7′), 31.8 (C6′), 45.6 (C5′), 50.5 (C1′), 55.1 (C2′), 61.5 (C8a′), 71.6 (C3, C-spiro), 110.4 (*d*, *J*_C–F_ = 6.9 Hz, CH), 113.5 (*d*, *J*_C–F_ = 24.7 Hz, CH), 116.0 (*d*, *J*_C–F_ = 23.6 Hz, CH), 127.6 (CH), 129.0 (CH), 129.4 (*d*, *J*_C–F_ = 7.4 Hz, C), 133.8 (CH), 136.9 (C), 139.0 (C), 159.5 (C), 173.4 (COOH), 179.3 (C2), 197.4 (C—CO—C). MS (EI, 70 eV) *m*/*z* (%): 408 (*M* ^+^, 10), 368 (17), 336 (37), 313 (12), 275 (36), 259 (16), 231 (39), 141 (22), 105 (49), 77 (34).

Compound (II): yield 48%; m.p. 529–530 K. Analysis found (%): C 71.2, H 5.9, N 7.0; calculated for C_24_H_24_N_2_O_4_ (%): C 71.3, H 6.0, N 6.9. FT–IR (ATR, cm^−1^): 3364, 3225, 2957, 1741, 1704, 1668. NMR (DMSO-*d*_6_): δ(^1^H) 1.10–1.20 (*m*, 2H, H8′, H7′), 1.21–1.32 (*m*, 1H, H6′), 1.43–1.50 (*m*, 1H, H7′), 1.67–1.74 (*m*, 1H, H8′), 2.05–2.18 (*m*, 5H, 5-CH_3_, H5′, H6′), 2.23 (*td*, *J* = 10.9, 2.8 Hz, 1H, H5′), 3.22–3.29 (*m*, 1H, H8a′), 3.44 (*t*, *J* = 9.8 Hz, 1H, H1′), 4.47 (*d*, *J* = 9.6 Hz, 1H, H2′), 6.30 (*d*, *J* = 7.8 Hz, 1H, H7), 6.57 (*s*, 1H, H4), 6.71 (*d*, *J* = 7.8 Hz, 1H, H6), 7.28 (*t*, *J* = 7.7 Hz, 2H, H_*m*_), 7.38 (*d*, *J* = 7.3 Hz, 2H, H_o_), 7.43 (*t*, *J* = 7.3 Hz, 1H, H_*p*_), 10.35 (*s*, 1H, NH), 12.62 (*s*, 1H, COOH); δ(^13^C) 21.0 (5-CH_3_), 23.8 (C8′), 25.5 (C7′), 31.8 (C6′), 45.6 (C5′), 50.5 (C1′), 54.9 (C2′), 61.4 (C8a′), 71.4 (C-spiro), 109.1 (CH, C7), 126.7 (CH, C4), 127.5 (C), 127.6 (CH, C_*o*_), 128.8 (CH, C_*m*_), 129.7 (CH, C6), 130.9 (C), 133.4 (CH, C_*p*_), 137.2 (C), 140.3 (C), 173.6 (COOH), 179.3 (C2), 197.6 (C—CO—C). MS (EI, 70 eV) *m*/*z* (%): 404 (*M* ^+^, 9), 368 (5), 332 (39), 315 (15), 271 (30), 255 (17), 227 (69), 141 (33), 105 (100), 77 (69).

Compound (III): yield 49%; m.p. 492–493 K. Analysis found (%): C 71.3, H 5.9, N 6.9; calculated for C_24_H_24_N_2_O_4_ (%): C 71.3, H 6.0, N 6.9. FT–IR (ATR, cm^−1^): 2941, 2356, 1709, 1677. NMR (DMSO-*d*_6_): δ(^1^H) 1.09–1.21 (*m*, 2H, H8′, H7′), 1.21–1.36 (*m*, 1H, H6′), 1.38–1.52 (*m*, 1H, H7′), 1.66–1.81 (*m*, 1H, H8′), 2.01–2.18 (*m*, 2H, H5′, H6′), 2.23 (*t*, *J* = 10.2 Hz, 1H, H5′), 2.98 (*s*, 3H, N—CH_3_), 3.22–3.29 (*m*, 1H, H8a′), 3.42 (*t*, *J* = 10.0 Hz, 1H, H1′), 4.47 (*d*, *J* = 9.8 Hz, 1H, H2′), 6.52 (*d*, *J* = 7.8 Hz, 1H), 6.79–6.90 (*m*, 2H), 7.01 (*t*, *J* = 7.5 Hz, 1H), 7.20–7.31 (*m*, 4H), 7.37–7.47 (*m*, 1H), 12.66 (*s*, 1H, COOH); δ(^13^C) 23.7 (C8′), 25.4 (C7′), 26.2 (CH_3_), 31.8 (C6′), 45.6 (C5′), 50.4 (C1′), 55.9 (C2′), 61.8 (C8a′), 71.1 (C3, C-spiro), 108.2 (CH), 122.9 (CH), 125.6 (CH), 127.0 (C), 127.4 (CH), 128.7 (CH), 129.6 (CH), 133.5 (CH), 137.0 (C), 144.0 (C), 173.4 (COOH), 177.4 (C2), 197.3 (C—CO—C). MS (EI, 70 eV) *m*/*z* (%): 404 (*M* ^+^, 1), 393 (12), 368 (22), 339 (26), 313 (70), 264 (34), 236 (16), 57 (100).

Compound (IV): yield 69%; m.p. 497–497 K. Analysis found (%): C 65.6, H 5.2, N 6.4; calculated for C_24_H_23_ClN_2_O_4_ (%): C 65.7, H 5.3, N 6.4. FT–IR (ATR, cm^−1^): 3378, 3227, 2957, 1744, 1708, 1668. NMR (DMSO-*d*_6_): δ(^1^H) 1.11–1.22 (*m*, 2H), 1.22–1.36 (*m*, 1H), 1.40–1.50 (*m*, 1H), 1.66–1.79 (*m*, 1H), 2.05–2.19 (*m*, 2H), 2.19–2.29 (*m*, 1H), 2.97 (*s*, 3H, CH_3_), 3.24 (*td*, *J* = 10.2, 2.5 Hz, 1H, H8a′), 3.43 (*t*, *J* = 10.0 Hz, 1H, H1′), 4.48 (*d*, *J* = 9.9 Hz, 1H, H2′), 6.56 (*d*, *J* = 8.3 Hz, 1H), 6.79 (*d*, *J* = 2.1 Hz, 1H), 7.09 (*dd*, *J* = 8.3, 2.2 Hz, 1H), 7.23–7.35 (*m*, 4H), 7.45 (*td*, *J* = 7.0, 1.6 Hz, 1H); δ(^13^C) 23.6 (C8′), 25.4 (C7′), 26.4 (CH_3_), 31.7 (C6′), 45.7 (C5′), 50.2 (C1′), 56.1 (C2′), 62.0 (C8a′), 71.1 (C3, C-spiro), 109.9 (CH), 125.5 (CH), 127.0 (C), 127.4 (CH), 128.9 (CH), 129.2 (C), 129.5 (CH), 133.8 (CH), 136.8 (C), 142.9 (C), 173.3 (COOH), 177.0 (C2), 197.3 (C—CO—C). MS (EI, 70 eV) *m*/*z* (%): 438 (*M* ^+^, 1), 336 (17), 313 (18), 275 (17), 231 (30), 141 (41), 105 (91), 77 (55), 57 (87), 43 (100).

Compound (V): yield 48%; m.p. 449–450 K. Analysis found (%): C 73.4, H 7.2, N 5.9; calculated for C_29_H_34_N_2_O_4_ (%): C 73.4, H 7.2, N 5.9. FT–IR (ATR, cm^−1^): 2931, 2858, 1723, 1684, 1662. NMR (DMSO-*d*_6_): δ(^1^H) 0.79–0.91 (*m*, 3H, CH_3_), 1.08–1.22 (*m*, 2H), 1.20–1.33 (*m*, 7H), 1.35–1.57 (*m*, 3H), 1.66–1.78 (*m*, 1H), 2.00–2.10 (*m*, 1H), 2.11–2.27 (*m*, 2H), 3.23–3.29 (*m*, 1H, H8a′), 3.37–3.51 (*m*, 2H, NCHH, H1′), 3.60 (*dt*, *J* = 14.5, 7.4 Hz, 1H, NCHH), 4.48 (*d*, *J* = 9.7 Hz, 1H, H2′), 6.60 (*d*, *J* = 7.8 Hz, 1H), 6.74–6.91 (*m*, 2H), 7.01 (*t*, *J* = 7.5 Hz, 1H), 7.19–7.32 (*m*, 4H), 7.42 (*t*, *J* = 7.2 Hz, 1H), 12.68 (*s*, 1H, COOH); δ(^13^C) 14.3 (CH_3_), 22.5 (CH_2_), 23.7 (C8′), 25.5 (C7′), 26.4 (CH_2_), 27.4 (CH_2_), 31.3 (CH_2_), 31.8 (C6′), 39.7 (CH_2_), 45.5 (C5′), 50.5 (C1′), 55.4 (C2′), 61.6 (C8a′), 70.9 (C3, C-spiro), 108.4 (CH), 122.7 (CH), 126.0 (CH), 126.9 (C), 127.5 (CH), 128.8 (CH), 129.6 (CH), 133.5 (CH), 143.5 (C), 173.5 (COOH), 177.2 (C2), 197.4 (C—CO—C). MS (EI, 70 eV) *m*/*z* (%): 475 (*M* ^+^ + H, 3), 368 (22), 339 (35), 313 (75), 264 (39).

### Refinement   

Crystal data, data collection and refinement details are summarized in Table 1 ▸. The crystallographic atom labelling followed the convention employed previously (Quiroga *et al.*, 2017 ▸; Romo *et al.*, 2020 ▸). For com­pound (II), five low-angle reflections which had been attenuated by the beam stop (101, 111, 0
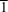
1, 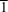
02 and 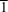
03) were omitted from the data set. All H atoms were located in difference maps. H atoms bonded to C atoms were then treated as riding atoms in geometrically idealized positions, with C—H = 0.95 (aromatic), 0.98 (CH_3_), 0.99 (CH_2_) or 1.00 Å (aliphatic C—H) and *U*_iso_(H) = *kU*_eq_(C), where *k* = 1.5 for the methyl groups, which were permitted to rotate but not to tilt, and 1.2 for all other H atoms bonded to C atoms. For the H atoms bonded to N or O atoms, the atomic coordinates were refined with *U*_iso_(H) = 1.2*U*_eq_(N) or 1.5*U*_eq_(O), giving the N—H and O—H distances shown in Table 2 ▸.

## Results and discussion   

All of the signals for the H and C atoms in com­pounds (I)–(V) were observed in their NMR spectra, with the sole exception of the carboxyl H-atom signal in com­pound (IV). All of the signals were assigned using one-dimensional spectra and two-dimensional COSY, HSQC and HMBC spectra. In terms of the formation of the spiro ring system, it is necessary to consider the NMR spectra only for com­pound (I), as those for (II)–(V) follow very similar lines, apart from the obvious differences arising from the differences in the peripheral substituents. The signals from atoms H1′ and H2′, bonded to atoms C1′ and C2′ (C21 and C22 in the crystallographic numbering scheme; see Fig. 1 ▸) which originated in the electron-deficient alkene, show a mutual coupling of 9.7 Hz, while H1′ is similarly coupled to H8a′, bonded to C8a′ (C28*A*). These signals confirm the formation of the new ring and the magnitude of the coupling constants show (Karplus, 1959 ▸) that atom H1′ is *trans* to both H2′ and H8′, so establishing the relative stereochemistry at atoms C1′, C2′ and C8a′ (C21, C22 and C28*A*). However, the NMR data do not allow definition of the stereochemistry of the spiro C atom relative to these three centres, nor that of the relative location of the benzoyl and carbonyl substituents, both of which were determined from the single-crystal diffraction study.

Compounds (I) and (II) are isomorphous, as are com­pounds (III) and (IV) (Table 1 ▸). Each com­pound contains four contiguous stereogenic centres, at atoms C21, C22, C13 and C28*A* (Figs. 1 ▸–5 ▸ ▸ ▸ ▸), and the centrosymmetric space groups (Table 1 ▸) confirm that each com­pound has crystallized as a racemic mixture. For each com­pound, the reference mol­ecule was selected as one having the *R* configuration at atom C13; on this basis, the configurations at atoms C21, C22 and C28*A* are *S*, *S* and *R*, respectively, with these atoms corresponding to locants C3, C1′, C2′ and C8a′ in the chemical numbering scheme, so that the overall configuration in each of (I)–(V) is (1′*SR*,2′*SR*,3*RS*,8a′*RS*). The structure analyses also show that for each com­pound, the carboxyl group is bonded to atom C21 and the benzoyl group is bonded to atom C22 (Figs. 1 ▸–5 ▸ ▸ ▸ ▸).

A plausible mechanism for the formation of com­pounds (I)–(V), based on previous work (Pardasani *et al.*, 2003 ▸; Quiroga *et al.*, 2017 ▸; Romo *et al.*, 2020 ▸), involves a condensation reaction between a substituted isatin (*A*) (Scheme 2) and pipecolic acid (*B*) to give inter­mediate (*C*), followed by dehydration to (*D*) and deca­rboxylation to give the ylide (*E*). The subsequent reaction between ylide (*E*) and *trans*-3-ben­zoyl­acrylic acid (*F*), neither of which contains any stereogenic centres, is both regio- and stereo­specific. Compounds (I)–(V) were all formed as racemic mixtures of a single stereoisomer, and formation of the alternative regioisomers of type (*G*) was not detected in any of the reactions. The *endo* approach of the alkene to the ylide is preferred over the alternative *exo* approach, as its transition state is better stabilized by π–π inter­actions between the aryl groups in the two reaction com­ponents.

The synthetic pathway defined in Scheme 2 thus significantly amplifies the scope of the ylide/alkene route to novel spiro com­pounds. The product yields, which are com­parable with, say, those of a three-step process with conversions of 80–85% at each stage, are regarded as entirely acceptable in view of the one-step nature of the procedure, the ready availability of starting materials which permit a very wide range of substituent combinations, and the regio- and stereo­specificity giving racemic mixtures of single stereoisomers.

The conformations (Evans & Boeyens, 1989 ▸) of the five-membered ring containing atom N24 show some unexpected variations. Thus, in the isomorphous pair (I) and (II), this ring adopts a half-chair conformation in (I), but an envelope conformation in (II). In (I), the ring is twisted about a line between atom C22 and the mid-point of the N24—C28*A* bond, such that atoms C13 and C21 are displaced to either side of the plane through atoms C22, N24 and C28*A* by 0.5324 (18) and 0.6374 (15) Å, respectively. In contrast, the corresponding ring in (II) adopts an envelope conformation, with the ring folded across the C21⋯N24 line and with atom C28*A* displaced by 0.6528 (19) Å from the plane through atoms C21, C22, C13 and N24. Similarly, in the isomorphous pair (III) and (IV), this ring adopts a half-chair conformation, but now twisted across the line between atom C13 and the mid-point of the C21—C28*A* bond, with atoms C22 and C24 displaced to either side of the plane through C13, C21 and C28*A* by 0.5410 (18) and 0.5819 (16) Å, respectively, while in (IV), this ring adopts the envelope conformation, folded across the C21⋯C24 line, with atom C28*A* displaced by 0.6709 (16) Å from the plane of the other four atoms. The same envelope conformation is found in (V), with a displacement of 0.6284 (19) Å for atom C28*A*. In each of (I)–(V), the six-membered ring containing atom N24 adopts an almost perfect chair conformation, with substituents C13 and C21 both in equatorial sites. The values of the ring-puckering parameters (Cremer & Pople, 1975 ▸; Boeyens, 1978 ▸) are summarized in Table 3 ▸. In view of the conformational differences within the isomorphous pairs (I)/(II) and (III)/(IV), it may not be appropriate to regard these pairs as strictly isostructural (Acosta *et al.*, 2009 ▸; Blanco *et al.*, 2012 ▸).

**Figure 6:**
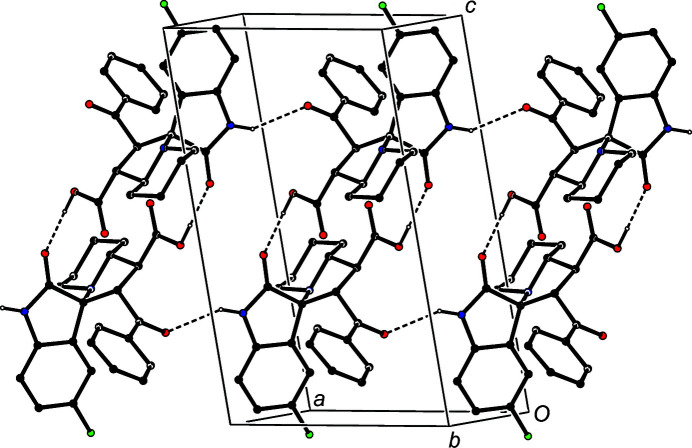
Part of the crystal structure of com­pound (I), showing the formation of a chain of rings along [100] built from O—H⋯O and N—H⋯O hydrogen bonds. For the sake of clarity, H atoms bonded to C atoms have all been omitted.

**Figure 7:**
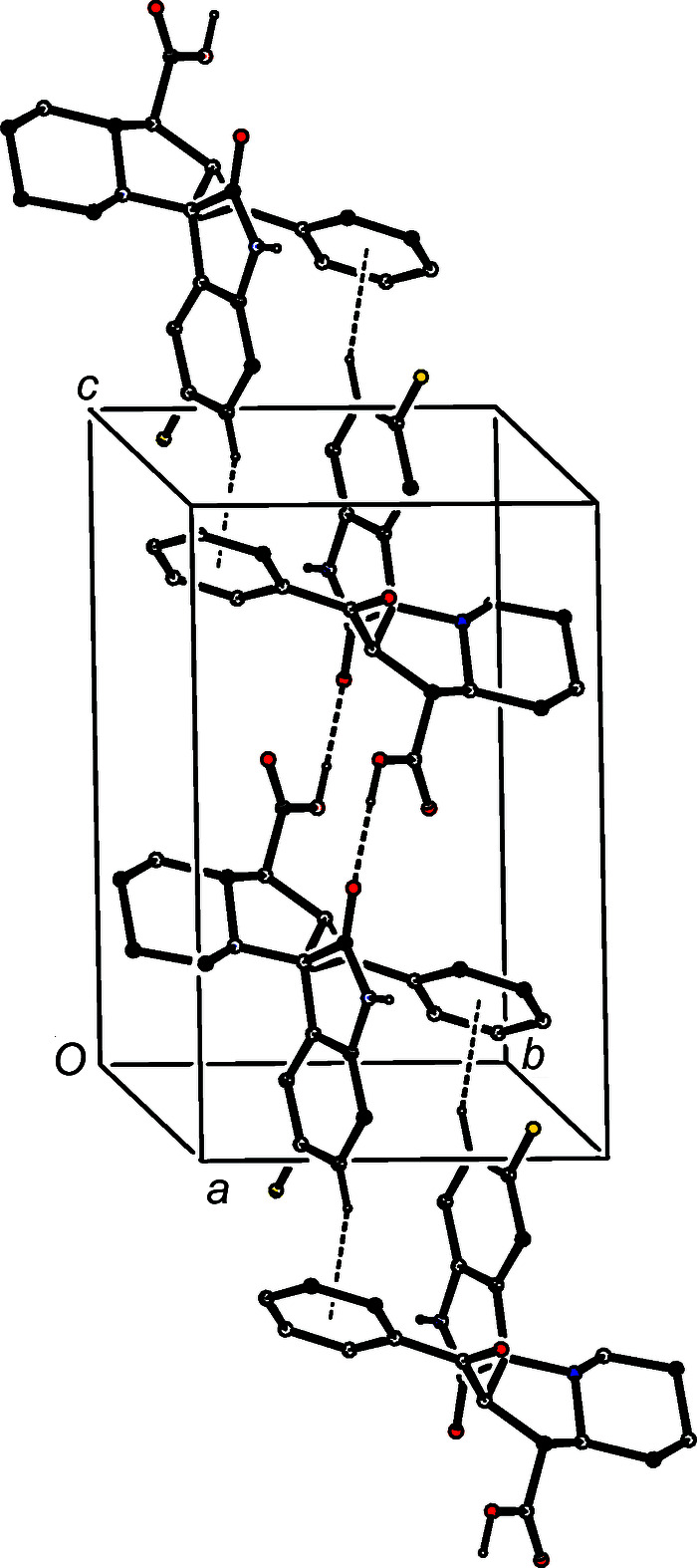
Part of the crystal structure of com­pound (I), showing the formation of a chain of rings along [10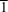] built from O—H⋯O and C—H⋯π(arene) hydrogen bonds. For the sake of clarity, H atoms bonded to those C atoms not involved in the motif shown have been omitted.

In the structure of com­pound (I), four hydrogen bonds (Table 2 ▸) link the mol­ecules into com­plex sheets whose formation can, however, be readily analysed in terms of two one-dimensional substructures (Ferguson *et al.*, 1998*a* ▸,*b* ▸; Gregson *et al.*, 2000 ▸). A combination of O—H⋯O and N—H⋯O hydrogen bonds forms a ribbon in the form of a chain of edge-fused centrosymmetric rings running parallel to [100], in which 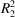
(16) (Etter, 1990 ▸; Etter *et al.*, 1990 ▸; Bernstein *et al.*, 1995 ▸) rings centred at (*n* + 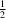, 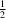, 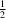) alternate with 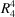
(22) rings centred at (*n*, 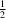, 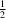), where *n* represents an integer in each case (Fig. 6 ▸). The formation of this ribbon is modestly enhanced by a C—H⋯O hydrogen bond involving a C—H bond of rather low acidity. In the second substructure, a combination of O—H⋯O and C—H⋯π(arene) hydrogen bonds forms a second chain of rings, this time running parallel to [10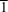], in which 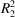
(16) rings centred at (*n* + 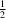, 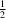, −*n* + 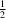) alternate with rings formed by the C—H⋯π(arene) hydrogen bonds, which are centred at (*n*, 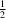, 1 − *n*), where *n* represents an integer in each case (Fig. 7 ▸). The combination of these two chain motifs generates a sheet lying parallel to (101), but there are no direction-specific inter­actions between adjacent sheets. The supra­molecular assembly of the isomorphous com­pound (II) is entirely similar to that in (I).

**Figure 8:**
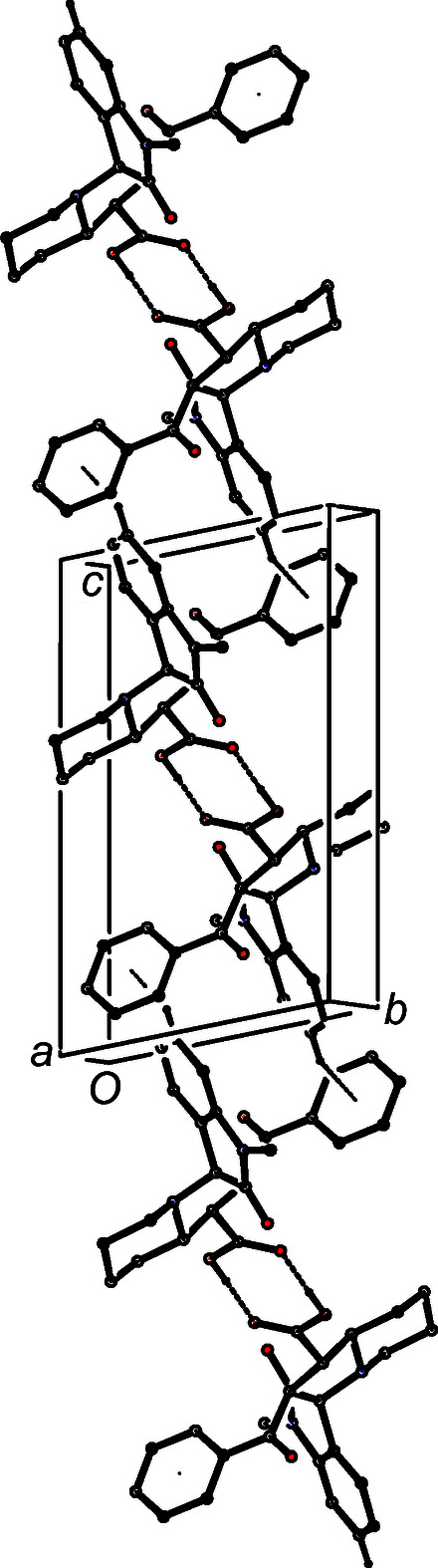
Part of the crystal structure of com­pound (III), showing the formation of a chain of rings along [101] built from O—H⋯O and C—H⋯π(arene) hydrogen bonds. For the sake of clarity, H atoms not involved in the motif shown have been omitted.

In the isomorphous pair of com­pounds (III) and (IV), there are just two hydrogen bonds (Table 2 ▸), and these link the mol­ecules into a chain of centrosymmetric rings running parallel to [101], in which 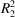
(8) rings formed by the O—H⋯O hydrogen bonds and centred at (*n* + 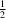, 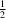, *n* + 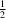) alternate with rings formed by C—H⋯π(arene) hydrogen bonds and centred at (*n*, 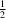, *n*), where *n* represents an integer in each case (Fig. 8 ▸). There are no direction-specific inter­actions between adjacent chains.

**Figure 9:**
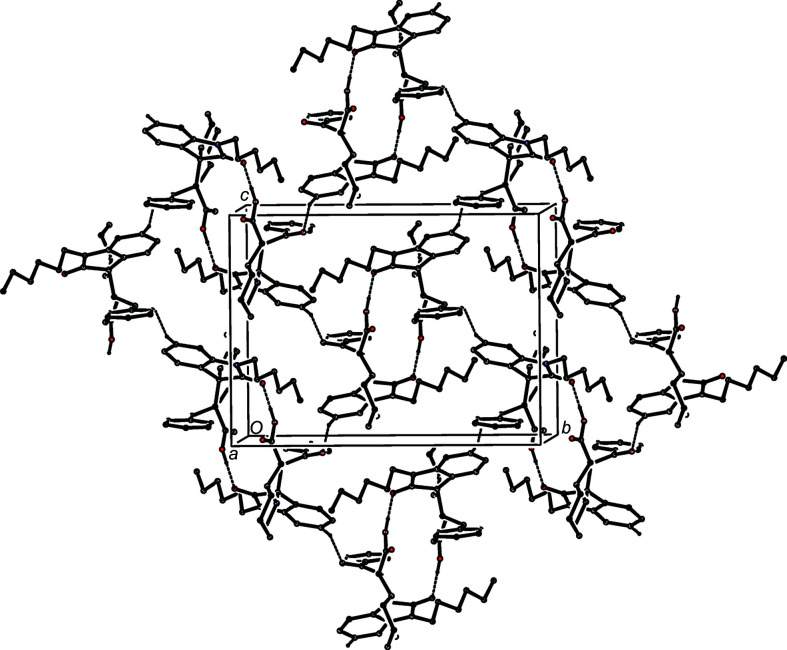
Part of the crystal structure of com­pound (V), showing the formation of a sheet lying parallel to (10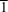) built from O—H⋯O and C—H⋯O hydrogen bonds. For the sake of clarity, H atoms bonded to those C atoms not involved in the motif shown have been omitted.

Five hydrogen bonds (Table 2 ▸) link the mol­ecules of com­pound (V) into sheets lying parallel to (10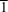), but the formation of the sheet can, in fact, be analysed in terms of just two of these inter­actions, those having atoms O212 and C16 as the donors. Inversion-related pairs of mol­ecules are linked by paired O—H⋯O hydrogen bonds to form centrosymmetric 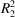
(16) dimers, of the type seen also in com­pounds (I) and (II), although in (V) the dimer formation is weakly augmented by two C—H⋯O inter­actions. Linkage of these dimers by the C—H⋯O hydrogen bond involving atom C16 then generates a sheet in which centrosymmetric rings of 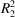
(16) and 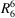
(46) types alternate in a chessboard fashion (Fig. 9 ▸). There are no direction-specific inter­actions between adjacent sheets.

Overall, therefore, the supra­molecular assembly is one-dimensional in each of com­pounds (III) and (IV), and two-dimensional in (I), (II) and (V); however, a three-dimensional assembly is not observed amongst the examples reported here. This may be contrasted with the behaviour observed in two spiro­[indoline-3,3′-pyrrolizine]s (Quiroga *et al.*, 2017 ▸). In (1′*RS*,2′*RS*,3*SR*,7a′*RR*)-1′,2′-bis­(4-chloro­benzo­yl)-5,7-di­chloro-2-oxo-1′,2′,5′,6′,7′,7a′-hexa­hydro­spiro­[indoline-3,3′-pyrrolizine], which crystallizes as a partial di­chloro­methane solvate, the heterocyclic mol­ecules are linked by N—H⋯O hydrogen bonds to form 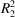
(8) dimers, while in (1′*RS*,2′*RS*,3*SR*,7a′*SR*)-2′-benzoyl-1-hexyl-2-oxo-1′,2′,5′,6′,7′,7a′-hexa­hydro­spiro­[indo­line-3,3′-pyrrolizine]-1′-carb­oxy­lic acid, the mol­ecules are linked by O—H⋯O hydrogen bonds to form cyclic 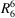
(48) hexa­mers with 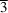 (*S*_6_) symmetry, which are further linked by C—H⋯O hydrogen bonds to form a three-dimensional framework structure.

In summary, therefore, we have developed a new application of the ylide/alkene procedure, which we have now used for the formation of spiro­[indoline-3,3′-indolizine]s in a single step, using simple and readily available starting materials. This approach permits the incorporation of a wide variety of substituents and other functional groups for further elaboration. Five representative examples have been fully characterized spectroscopically and structurally, and their patterns of supra­molecular assembly have been analysed, described and illustrated.

## Supplementary Material

Crystal structure: contains datablock(s) global, I, II, III, IV, V. DOI: 10.1107/S2053229621007142/ky3207sup1.cif

Structure factors: contains datablock(s) I. DOI: 10.1107/S2053229621007142/ky3207Isup2.hkl

Structure factors: contains datablock(s) II. DOI: 10.1107/S2053229621007142/ky3207IIsup3.hkl

Structure factors: contains datablock(s) III. DOI: 10.1107/S2053229621007142/ky3207IIIsup4.hkl

Structure factors: contains datablock(s) IV. DOI: 10.1107/S2053229621007142/ky3207IVsup5.hkl

Structure factors: contains datablock(s) V. DOI: 10.1107/S2053229621007142/ky3207Vsup6.hkl

Supporting information file. DOI: 10.1107/S2053229621007142/ky3207Isup7.cml

Supporting information file. DOI: 10.1107/S2053229621007142/ky3207IIsup8.cml

Supporting information file. DOI: 10.1107/S2053229621007142/ky3207IIIsup9.cml

Supporting information file. DOI: 10.1107/S2053229621007142/ky3207IVsup10.cml

Supporting information file. DOI: 10.1107/S2053229621007142/ky3207Vsup11.cml

CCDC references: 2095737, 2095736, 2095735, 2095734, 2095733

## Figures and Tables

**Figure 2:**
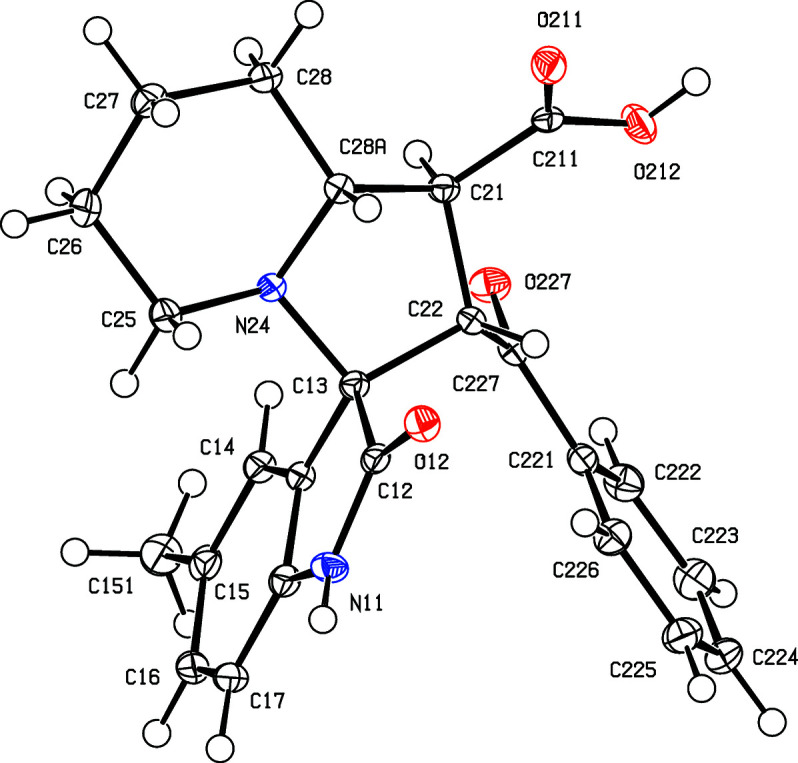
The mol­ecular structure of the (1′*S*,2′*S*,3*RS*,8a′*R*) enanti­omer of compound (II), showing the atom-labelling scheme. Displacement ellipsoids are drawn at the 50% probability level.

**Figure 3:**
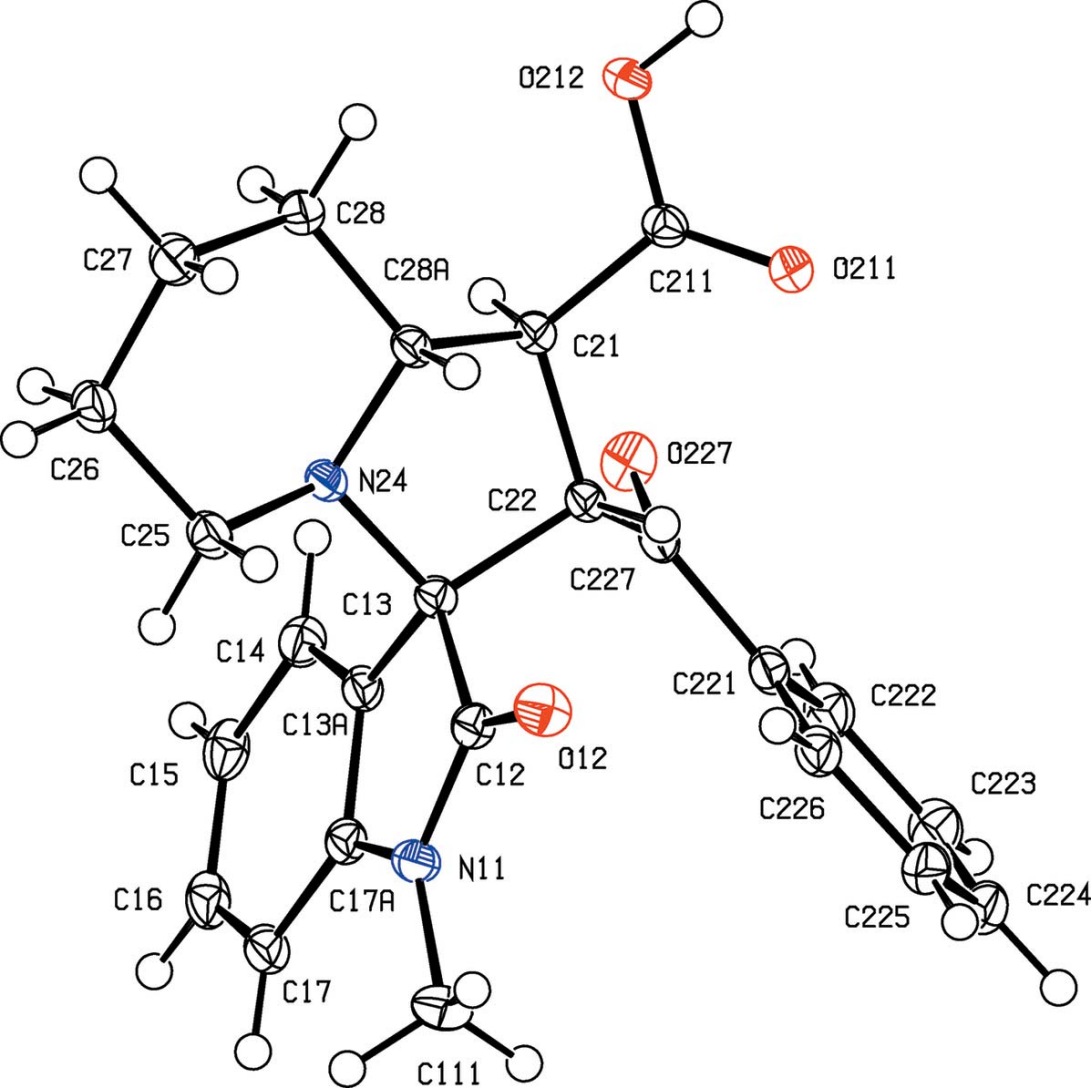
The mol­ecular structure of the (1′*S*,2′*S*,3*RS*,8a′*R*) enanti­omer of compound (III), showing the atom-labelling scheme. Displacement ellipsoids are drawn at the 50% probability level.

**Figure 4:**
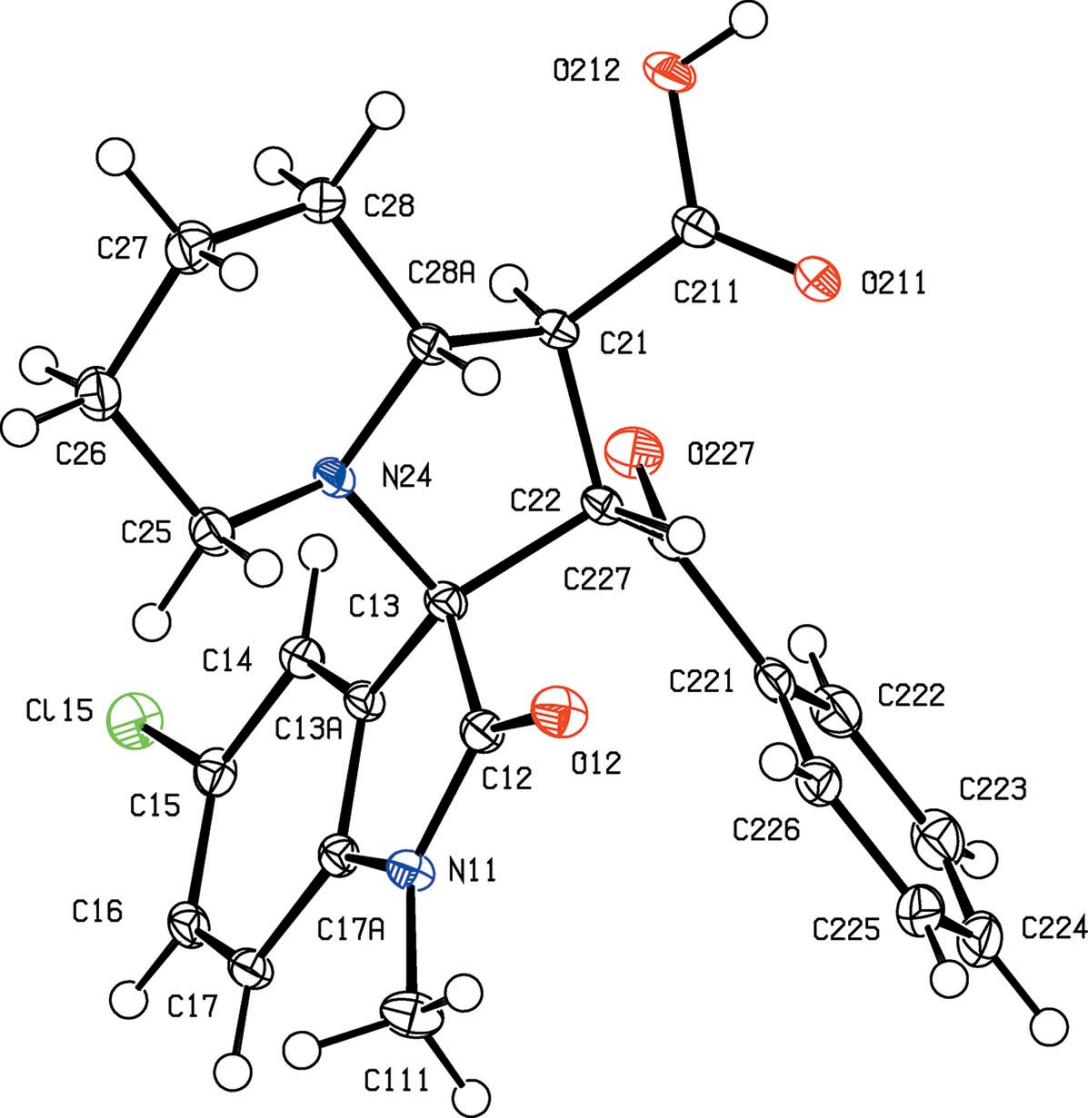
The mol­ecular structure of the (1′*S*,2′*S*,3*RS*,8a′*R*) enanti­omer of compound (IV), showing the atom-labelling scheme. Displacement ellipsoids are drawn at the 50% probability level.

**Figure 5:**
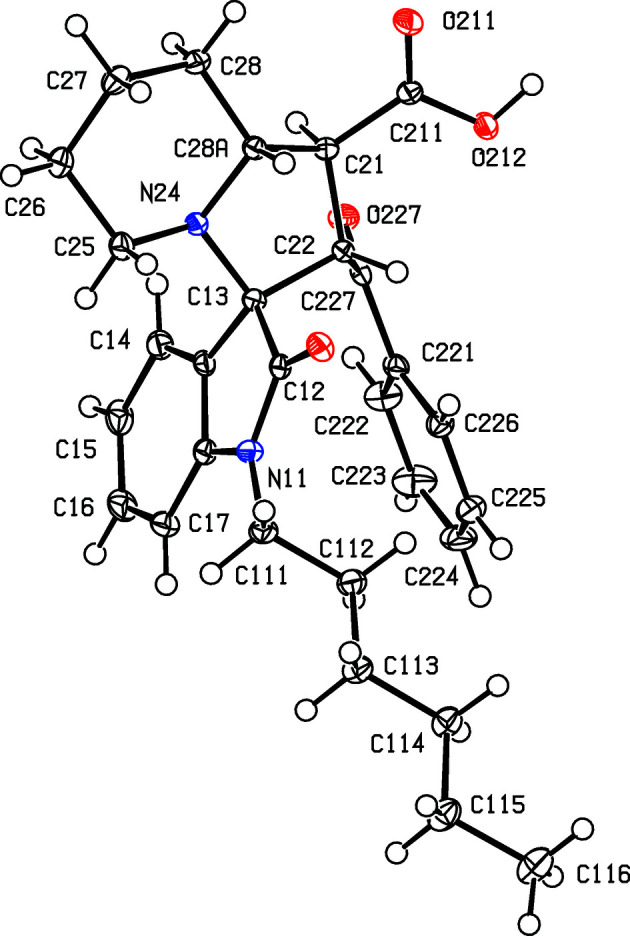
The mol­ecular structure of the (1′*S*,2′*S*,3*RS*,8a′*R*) enanti­omer of compound (V), showing the atom-labelling scheme. Displacement ellipsoids are drawn at the 50% probability level.

**Table:** Experiments were carried out at 100 K with Mo *K*α radiation using a Bruker D8 Venture diffractometer. Absorption was corrected for by multi-scan methods (*SADABS*; Bruker, 2016 ▸). H atoms were treated by a mixture of independent and constrained refinement.

	(I)	(II)	(III)
Crystal data			
Chemical formula	C_23_H_21_FN_2_O_4_	C_24_H_24_N_2_O_4_	C_24_H_24_N_2_O_4_
*M*_r_	408.42	404.45	404.45
Crystal system, space group	Triclinic, *P*\overline{1}	Triclinic, *P*\overline{1}	Triclinic, *P*\overline{1}
*a*, *b*, *c* (Å)	8.1440 (5), 8.4565 (5), 14.9945 (8)	8.1874 (6), 8.5015 (6), 15.5775 (12)	8.6535 (4), 9.2064 (4), 14.4327 (6)
α, β, γ (°)	87.549 (2), 79.926 (2), 68.467 (2)	85.775 (3), 77.641 (3), 68.022 (2)	72.660 (1), 74.539 (1), 65.930 (2)
*V* (Å^3^)	945.52 (10)	982.15 (13)	988.16 (8)
*Z*	2	2	2
μ (mm^−1^)	0.11	0.09	0.09
Crystal size (mm)	0.26 × 0.21 × 0.12	0.16 × 0.12 × 0.07	0.19 × 0.19 × 0.12

Data collection			
*T*_min_, *T*_max_	0.939, 0.987	0.934, 0.993	0.944, 0.989
No. of measured, independent and observed [*I* > 2σ(*I*)] reflections	50206, 4727, 3889	40953, 4490, 3667	47689, 4917, 4145
*R*_int_	0.068	0.069	0.057
(sin θ/λ)_max_ (Å^−1^)	0.668	0.650	0.667

Refinement			
*R*[*F*^2^ > 2σ(*F* ^2^)], *wR*(*F* ^2^), *S*	0.037, 0.095, 1.04	0.040, 0.098, 1.03	0.039, 0.098, 1.04
No. of reflections	4727	4490	4917
No. of parameters	277	278	275
Δρ_max_, Δρ_min_ (e Å^−3^)	0.32, −0.26	0.34, −0.22	0.30, −0.33

**Table:** 

	(IV)	(V)
Crystal data		
Chemical formula	C_24_H_23_ClN_2_O_4_	C_29_H_34_N_2_O_4_
*M*_r_	438.89	474.58
Crystal system, space group	Triclinic, *P*\overline{1}	Monoclinic, *P*2_1_/*n*
*a*, *b*, *c* (Å)	8.7914 (9), 9.3155 (10), 14.6188 (15)	11.0442 (4), 17.4707 (6), 13.0081 (4)
α, β, γ (°)	73.437 (4), 76.259 (4), 64.156 (3)	90, 90.215 (1), 90
*V* (Å^3^)	1023.84 (19)	2509.89 (15)
*Z*	2	4
μ (mm^−1^)	0.22	0.08
Crystal size (mm)	0.41 × 0.32 × 0.14	0.23 × 0.13 × 0.12

Data collection		
*T*_min_, *T*_max_	0.934, 0.969	0.921, 0.990
No. of measured, independent and observed [*I* > 2σ(*I*)] reflections	41790, 5097, 4493	24313, 5765, 4619
*R*_int_	0.055	0.059
(sin θ/λ)_max_ (Å^−1^)	0.668	0.650

Refinement		
*R*[*F*^2^ > 2σ(*F* ^2^)], *wR*(*F* ^2^), *S*	0.033, 0.081, 1.03	0.043, 0.102, 1.03
No. of reflections	5097	5765
No. of parameters	284	320
Δρ_max_, Δρ_min_ (e Å^−3^)	0.33, −0.31	0.29, −0.26

Computer programs: *APEX3* (Bruker, 2018 ▸), *SAINT* (Bruker, 2017 ▸), *SHELXT2014* (Sheldrick, 2015*a* ▸), *SHELXL2014* (Sheldrick, 2015*b* ▸) and *PLATON* (Spek, 2020 ▸).

**Table 2:** Hydrogen-bond parameters (Å, °) *Cg*1 represents the centroid of the C221–C226 ring.

	*D*—H⋯*A*	*D*—H	H⋯*A*	*D*⋯*A*	*D*—H⋯*A*
(I)	N11—H11⋯O227^i^	0.868 (18)	2.242 (17)	2.9756 (16)	142.2 (13)
	O212—H212⋯O12^ii^	0.899 (19)	1.873 (19)	2.7723 (14)	178.4 (15)
	C22—H22⋯O211^ii^	1.00	2.22	3.1930 (15)	164
	C16—H15⋯*Cg*1^iii^	0.95	2.63	3.4966 (14)	152
(II)	N11—H11⋯O227^i^	0.880 (18)	2.181 (18)	2.9598 (18)	147.3 (15)
	O212—H212⋯O12^ii^	0.89 (2)	1.88 (2)	2.7691 (15)	179 (2)
	C22—H22⋯O211^ii^	1.00	2.21	3.1894 (16)	165
	C16—H15⋯*Cg*1^iii^	0.95	2.75	3.6366 (16)	155
(III)	O212—H212⋯O211^ii^	0.920 (19)	1.798 (19)	2.7162 (14)	175.5 (16)
	C16—H16⋯*Cg*1^iv^	0.95	2.70	3.5181 (17)	144
(IV)	O212—H212⋯O211^ii^	0.863 (19)	1.854 (19)	2.7152 (14)	175.3 (16)
	C16—H16⋯*Cg*1^iv^	0.95	2.52	3.3872 (14)	152
(V)	O212—H212⋯O12^ii^	0.915 (18)	1.744 (18)	2.6589 (13)	178.5 (15)
	C113—H11*F*⋯O227^v^	0.99	2.51	3.4738 (17)	163
	C16—H16⋯O227^vi^	0.95	2.48	3.3947 (17)	162
	C22—H22⋯O211^ii^	1.00	2.57	3.5693 (16)	177
	C226—H226⋯O211^ii^	0.95	2.50	3.3854 (17)	155

Symmetry codes: (i) *x* + 1, *y*, *z*; (ii) −*x* + 1, −*y* + 1, −*z* + 1; (iii) −*x* + 2, −*y* + 1, −*z*; (iv) −*x*, −*y* + 1, −*z*; (v) −*x* + {1\over 2}, *y* + {1\over 2}, −*z* + {1\over 2}; (vi) *x* − {1\over 2}, −*y* + {1\over 2}, *z* − {1\over 2}.

**Table 3:** Ring-puckering parameters (Å, °) Parameters for rings *A* and *B* are calculated for the atom sequences N24—C13—C22—C21—C28*A* and N24—C25—C26—C27—C28—C28*A*, respectively.

Ring *A*	*Q*_2_	φ_2_	
(I)	0.4391 (11)	333.05 (17)	
(II)	0.4363 (14)	332.26 (19)	
(III)	0.4436 (13)	312.16 (17)	
(IV)	0.4456 (13)	317.67 (17)	
(V)	0.4125 (13)	327.81 (18)	
			
Ring *B*	*Q*	θ	φ
(I)	0.5673 (14)	175.99 (14)	227.3 (19)
(II)	0.5670 (15)	176.31 (15)	226 (2)
(III)	0.5846 (14)	176.53 (14)	141 (3)
(IV)	0.5913 (14)	176.98 (14)	132 (3)
(V)	0.5778 (14)	179.45 (14)	219 (22)
